# Lifestyle and Outcomes of Assisted Reproductive Techniques: A Narrative Review

**DOI:** 10.5539/gjhs.v7n5p11

**Published:** 2015-02-24

**Authors:** Hamzehgardeshi Zeinab, Shahhosseini Zohreh, Keshvar Samadaee Gelehkolaee

**Affiliations:** 1 Department of Reproductive Health and Midwifery, Nasibeh Nursing and Midwifery Faculty, Mazandaran University of Medical Sciences, Sari, Iran; 2 Traditional and Complementary Medicine Research Centre, Mazandaran University of Medical Sciences, Sari, Iran; 3 Student Research Committee, Mazandaran University of Medical Sciences, Sari, Iran

**Keywords:** lifestyle, assisted reproductive techniques, narrative review

## Abstract

**Background:**

Studies reveal that lifestyles such as physical activity patterns, obesity, nutrition, and smoking, are factors that affect laboratory test results and pregnancy outcomes induced by assisted fertility techniques in infertile couples. The present study is a narrative review of studies in this area.

**Methods:**

In this study, researchers conducted their computer search in public databases Google Scholar general search engine, and then more specific: Science Direct, ProQuest, SID, Magiran, Irandoc, Pubmed, Scopus, cochrane library, and Psych info; Cumulative Index to Nursing and Allied Health Literature (CINAHL), using Medical Subject Headings (MeSH) keywords: infertility (sterility, infertility), lifestyle (life behavior, lifestyle), Assisted Reproductive Techniques (ART), antioxidant and infertility, social health, spiritual health, mental health, Alcohol and drug abuse, preventive factors, and instruments., and selected relevant articles to the study subject from 2004 to 2013. Firstly, a list of 150 papers generated from the initial search. Then reviewers studied titles and abstracts. Secondly, 111 papers were included. Finally, quality assessment of full text studies was performed by two independent reviewers. Researchers reviewed summary of all articles sought, ultimately used data from 62 full articles to compile this review paper.

**Results:**

Review of literature led to arrangement of 9 general categories of ART results’ relationship with weight watch and diet, exercise and physical activity, psychological health, avoiding medications, alcohol and drugs, preventing diseases, environmental health, spiritual health, social health, and physical health.

**Conclusion:**

The following was obtained from review of studies: since lifestyle is among important, changeable, and influential factors in fertility, success of these methods can be greatly helped through assessment of lifestyle patterns of infertile couples, and design and implementation of healthy lifestyle counseling programs, before and during implementing assisted fertility techniques.

## 1. Introduction

Infertility means a couple in their reproductive age not bearing a child after a year’s unprotected intercourse. It has a global prevalence of 12% to 15% (McLaren, 2012). There are various factors involved including physical, psychological, and social, collectively known as lifestyle. Lifestyle involves attempt to achieve complete physical, psychological, and social well-being, and includes weight watch, exercise, diet, prevention of diseases, avoiding alcohol and drugs, mental health, spiritual health, social health, increased pregnancy age, and prevention of accidents (Krawczyk & Kalinowski, 2011; Lali, Abedi, & Kajbaf, 2012; Nakao, Honda, Moji, Abe, & Aoyagi, 2011).

Currently, the latest method of dealing with this problem is Assisted Reproductive Techniques (ART) that involves direct manipulation of oocytes and sperm in vitro, which has led to dramatic reduction in infertility rates (Anderson, Norman, & Middleton, 2010; Ebbesen et al., 2009). Success of these techniques is largely dependent upon lifestyle, and failure can impose heavy financial and psychological burden on the family and the society. Considering that few all-inclusive research has been conducted into associated factors with lifestyle (Anderson et al., 2010; Kazemi, Esfahani, Ahmadi, Ehsanpour, & Ganji, 2009). Thus, this review study aimed to identify an ideal lifestyle in assisted reproductive treatment cycle through review of relevant literature to infertile people’s lifestyle and its relationship with ART results.

## 2. Method

The current narrative review followed the five steps, which are: 1. identifying the research question; 2. Search methods for Identifying relevant studies; 3. study selection; 4. charting the data, collating, summarizing and 5. Reporting the results (Cowley et al., 2013).

### 2.1 Identifying the Research Question

Are lifestyle types related to outcomes of assisted reproductive techniques?

### 2.2 Search Methods for Identifying Relevant Studies

Researchers used Google Scholar general search engine, and then more specific: Science Direct, ProQuest, SID, Magiran, Irandoc, Pubmed, Scopus, cochrane library, and Psych info; Cumulative Index to Nursing and Allied Health Literature (CINAHL). Keywords based on Medical Subject Headings (MeSH) used to search resources for collection of data included: infertility (sterility, infertility), lifestyle (life behavior, lifestyle), Assisted Reproductive Techniques (ART), antioxidant and infertility, social health, spiritual health, mental health, Alcohol and drug abuse, preventive factors, and instruments. Selected relevant articles to the study subject were from 2004 to 2013.

### 2.3 Study Selection

A list of 150 papers generated from the initial search. Then reviewers studied titles and abstracts. Secondly, 111 papers were included. Finally, quality assessment of full text studies was performed by two independent reviewers. Researchers reviewed summary of all articles sought, ultimately used data from 62 full articles to compile this review paper. Researchers assessed for inclusion all titles and abstracts without language limitations in English and Persian language.

#### 2.3.1 Inclusion Criteria

We included papers that described the relation of lifestyle types with ART outcomes in infertile women.

#### 2.3.2 Exclusion Criteria

We excluded papers that described the relation of lifestyle types with pregnancy outcomes in fertile women.

### 2.4 Charting the Data, Collating, Summarizing

Data extracted were summarize in Table 1, Table 2.

**Table 1:** Effect of lifestyle on fertility and infertility in dimensions of (weight gain and nutrition, exercise, avoiding alcohol and drugs, and disease prevention)

Dimensions of lifestyle	Effect mechanism	Results	Number of articles	Counseling advise
Weight gain and nutrition	Use of supplements, folate, iron, fat, carbohydrate, protein, weight variations, eating disorder	Impact on ovarian response to gonadotropin, sperm morphology, nervous tube defects, erectile dysfunction oligomenorrhea and amenorrhea	15	Maintaining 20<BMI<25 Use of fruits and vegetables, use of unsaturated fat
Physical activity and exercise	Regular exercise, non-intensive exercise	Sense of well-being and physical health Due to calorie imbalance and production of free oxygen radicals, reduced fertilization, sperm and DNA damage	1	Advise to professional athletes, reduced intensity and duration of exercise to 3 times per week, for 45-60 minutes
Disease prevention	Antibody in the body, blood pressure control, blood sugar control, prevention of sexually transmitted diseases	Maternal and fetal health, preventing early miscarriage, preventing pelvic infection, and subsequent adhesions	5	Timely vaccination, avoiding high-risk sexual behaviors
Avoiding alcohol, drugs and medications	Increased free oxygen radicals, increased semen leukocytes, endocrine disorder, effect on ovarian reserves, sexual dysfunction, impaired uterus tube motility	Reduced quality of sperm and embryo, reduced fertilization, increased prolactine, early menopause, changes in corpus luteum and ovarian vessels, poor ovarian response to fertilization, stillbirth	17	Teaching healthy lifestyle, dealing with negative mood, daring skill, problem solving skill

**Table 2:** Effect of lifestyle on fertility and infertility in dimensions of (physical, mental, spiritual, and environmental health)

Lifestyle dimensions	Mechanism	Results	Number of articles	Counseling advice
Physical health	Chronic diseases increase with aging, infertility hormonal changes, imbalance between oxidants and antioxidants threaten health	Reduced fertilization, delayed pregnancy, lack of endometrial acceptance, preeclampsia, preterm labor, moles	13	Use of antioxidants, pregnancy at appropriate age
Psychological health	Increased stress, anxiety, and depression, increased schizophrenia	Impact on sperm density, sperm morphology, neurotransmitter changes due to hypothalamus dysfunction, reduced testosterone and LH	7	Problem-solving skills, coping with negative mood and anxiety, cognitive-behavioral interventions
Social health	Desire to talk with others, removing tension, establishing relationships and asking for help, essential source of compliance, increased resilience, increased satisfaction	Increased quality of semen, reduced stress, increased endometrial acceptance,	9	Teaching communication and life skills, problem-solving skills
Spiritual health	Sense of belonging to a superior force, reduced negative burden, and negative effects of disease, sense of purpose, treatment follow-up	Creating psychological health, reduced stress, improved quality of life	6	Development of spirituality, respect for beliefs of references
Environmental health	Contact with toxic materials, such as lead, methyl mercury, pesticides, radioactive materials	Effects on sperm morphology, chromosomal mutation, fetal abnormalities, restricted growth, stillbirth, preterm labor, miscarriage	4	Avoiding exposure to chemicals, observing principles of safety, wearing proper work uniform

### 2.5 Reporting the Results

Reporting data were nine categories

## 3. Results

Review of literature led to arrangement of nine categories, including: The relationship of ART outcomes with physical health; The relationship between ART results and weight control and diet; The relationship of ART outcomes with exercise and physical activity; The relationship of ART results with psychological health; The relationship of ART outcomes with avoiding medication, drugs and alcohol; The relationship of ART outcomes with disease prevention; The relationship of ART outcomes with environmental health; The relationship of ART outcomes with spiritual health; and The relationship of ART outcomes with social health (Tables 1 and 2).

## 4. Discussion

Generally, the results show that unhealthy lifestyle adversely affects production of follicular liquid oxidants, the number of oocytes, their quality and fertilization rate, quality of embryos and pregnancy rate.

### 4.1 The Relationship of ART Outcomes With Physical Health

Physical health is an important and influential factor on Assisted Reproductive Techniques’ outcomes. All issues discussed can affect physical health. Furthermore, aging can affect people’s health and ART outcomes. Considering that many couples postpone pregnancy because of education, it should be noted that fertility has peak and trough points in both men and women. Thus, age should be taken into account in assessment of couple’s fertility, so that infertility assessment should commence in people over 35 years of age within 6 months, and in people over 40 years of age, straight away (McLaren, 2012). In men, level of testosterone and semen parameters reduce with aging, so that beyond 35 years, volume and motility reduces and abnormal form increases, and after 40 years, its motility falls below 40% and its survival rate, below 50%. In men older than 45 years, likelihood of infertility increases to 4.6% for every 1 year’s aging, and increases to 12.5% for every two years aging. Moreover, with aging, men tend to consume more alcohol, and will have lower libido (Mukhopadhyay et al., 2010; Sharma, Biedenharn, Fedor, & Agarwal, 2013). In women, both quality and quantity of ovarian reserves reduce with aging. For different reasons (hereditary, medication, chemotherapy), some women may experience early ovarian failure and menopause younger than 40 years (Berlinguer et al., 2012; Van Zonneveld et al., 2003). Also, with aging, incidence of cardiovascular diseases, arteriosclerosis, and diabetes are more likely, and all are involved in causing infertility (Novak & Berek, 2012). Risk of uterus and tube infection (salpangitis) and adhesion increases with aging, which can cause infertility (McLaren, 2012). Chances of pregnancy may fall to 71% after the age of 30 years, and to 45% after 35 years of age. Risk of chromosomal diseases, such as fetal aneuploidy increases with aging, resulting in early miscarriage, reported 45.7% in women older than 35 years, and 34.8% in women younger than 35 years (Pan, Ma, Zhu, & Schultz, 2008).

Oxidative stress is a substance produced in the body in particular circumstance, and can adversely affect health and ART outcomes. In fact, this substance is the very free oxygen radicals with great affinity to combine, and can cause tissue damage. They can especially affect fertility and IVF results, and are influential in pathogenesis of diseases such as cancer, moles, birth defects, endometrial changes, embryo development, preeclampsia, and preterm labor, which can all ultimately lead to miscarriage and infertility. Normally, these substances are cleared, but in special circumstances, the body is unable to do so, factors that increase production of oxidative stress materials include improper nutrition void of fruits and vegetables, use of alcohol and smoking, or exposure to cigarette smoke, intense physical activity and exhaustion, stress, and use of some medications (Ashok Agarwal, Gupta, & Sharma, 2005; Tomey et al., 2007). Different types of oxidative stress include OH- and H2O, O2-. Normally, these are neutralized by defense mechanisms of the body including enzymes: catalase, superoxide desmotase, reductase, and glutathione peroxidase, and also non-enzymes, including: vitamins C, E, and A, pirovate, turin and hypoturin. Non-enzymatic antioxidants are placed around oocytes and ovarian follicular fluid and protect them against harm. Production of this substance can cause harm, leading to ART failure in both men and women (Ashok Agarwal, Said, Bedaiwy, Banerjee, & Alvarez, 2006; Combelles, Gupta, & Agarwal, 2009). Moreover, exposure to environmental pollutants can also increase oxidative stress and endanger individual’s health. Also, its harmful effects have been demonstrated in animal studies and assessments of IVF success. Benefits of using antioxidants in increasing ART success have also been shown (Ashok Agarwal, Aponte-Mellado, Premkumar, Shaman, & Gupta, 2012; Ruder, Hartman, & Goldman, 2009). Researches recommend consider to physical health and antioxidant balance before infertile treatment.

### 4.2 Relationship Between ART Results and Weight Control and Diet

A healthy diet with appropriate calorie creates optimal physical and psychological health (Homan, Davies, & Norman, 2007). Taking folic acid and some vitamins, especially group B vitamins reduce risk of fetal neural defects by 70%. It has also been seen that reduced folate can reduce ovarian response to internal gonadotropin, resulting in impaired ovulation. Excessive intake of vitamin A can cause congenital malformations (De-Regil, Fernández-Gaxiola, Dowswell, & Peña-Rosas, 2010; Gardiner et al., 2008).

In men, consumption of food rich in fiber, carbohydrates, folate, lycopene, and fruits and vegetables increase sperm quality, and low intake of protein and fat and ample antioxidants are beneficial for fertility. In special circumstances, Reactive Oxygen Species (ROS) are produced in the body, which are dissolved with diet rich in antioxidants such as vitamin C and beta-carotene and vitamin E (Tomey et al., 2007). Intake of vitamin E and selenium reduces concentration of melondialdehyde (MDA) and increases spermatozoa mobility. Furthermore, live birth rates increased in spouses of men receiving oral antioxidants. In women, consumption of vegetable protein instead of carbohydrates and animal protein and unsaturated fat rather than trans fat dramatically reduced risk of infertility of impaired ovulation. Women that use iron supplement and multivitamins experience 73% less infertility (Sharma et al., 2013). Consumption of unsaturated fats, fatty acid omega 3, pulses as source of carbohydrate, balanced use of fruits and vegetables, are recommended as a proper diet, to prevent coronary diseases (Hu & Willett, 2002). Impaired ovulation, making up for 18%-30% of infertility causes, is associated with diet including consumption of iron, carbohydrates (quality and quantity), proteins, vitamins, and fatty acid, and it can be prevented with a balanced diet (Chavarro, Rich-Edwards, Rosner, & Willett, 2007). Also, use of iron supplements and vitamins (3 times per week) can reduce risk of infertility through affecting ovarian function (Chavarro, Rich-Edwards, Rosner, & Willett, 2006, 2008b). People receiving Mediterranean diet prior to In Vitro Fertilization (IVF) and Intra Cytoplasmic Sperm Injection (ICSI) including ample vegetables, vegetable oil, pulses, and fish, experience an increase in blood folate and vitamin B6, and follicular fluid, which increase chances of successful fertility (Vujkovic, et al., 2010). Therefore, it is recommended that in infertility clinics, the first step be screening dietary behaviors in infertile couples to help fertility (Chavarro, Rich-Edwards, Rosner, & Willett, 2008a; Twigt et al., 2012). Assessment of proper diet and healthy lifestyle behaviors in low fertile couples can reduce short-term harmful behaviors and positively affect reproductive function and fertility results (Hammiche et al., 2011).

Abnormal weight is referred to 20>BMI>25 (Homan et al., 2007). In men, increased weight causes reduction in sperm concentration and mobility, and increased DNA damage in sperm, there is also a relationship between obesity and erectile dysfunction. Moreover, 96.5% of men presenting with metabolic syndrome may suffer excessive hormonal conversion of androgen into estrogen, which may affect other hormones such as lipitin and inhibin B, resulting in reduced sertoli cells and sperm production (Sharma et al., 2013).

Insulin resistance increases with gaining weight, resulting in greater risk of metabolic syndrome and diabetes. On the other hand, increased insulin level affects the ovary and reduces fertility rate, so that for every unit increase in maternal BMI chances of implantation, pregnancy and live birth progressively and significantly are reduced (Cunningham et al., 2010). Prenatal weight gain is reported to cause increased rate of cesarean section, macrosomia, pre-eclampsia, gestational diabetes, and urinary tract infection, resulting in further use of health services (Norman et al., 2004; Vals, Kiivet, & Leinsalu, 2013). Weight gain can cause early miscarriage in various ways including 3-fold likelihood of fetal neural tube defect (Cunningham et al., 2010; Sharma et al., 2013). Miscarriage may be due to lack of endometrial receptivity, since there is a negative relationship between weight gain and implantation. Weight gain can led to changes as increased insulin in follicular fluid and plasma triglyceride, C-Reaction Protein (CRP) changes, and reduced Sex Hormone Bonding Globulin (SHBG). These changes are probably reversible. It is said that by weight loss of 10.2 kg, 90% of those that suffer anovulation can begin to ovulate again. Diseases associated with eating can adversely affect menopause, fertility and maternal and fetal health. People that suffer from oligomenorrhoea and amenorrhea have 58% chance of irregular menopause. Nearly 20.7% of infertile women have eating disorders (Sharma et al., 2013). Hence, couples that seek reproduction are advised to have BMI between 20 and 25 (Gardiner et al., 2008).

### 4.3 The Relationship of ART Outcomes With Exercise and Physical Activity

Regular physical exercise can positively affect a person’s health. Every exercise hour per week can reduce risk of infertility by 5%. Men that exercise 3 times per week, for one hour each time are better in terms of sperm parameters, compared to those that exercise more and more professionally. Cycling more than 5 hours per week reduces sperm mobility and concentration. Intense exercise can adversely affect calorie balance in the body. In women athletes, frequency and duration of exercise are considered as important infertility factors. A study on people undergoing IVF showed that more than 4 hours exercise per week, one year before pregnancy, reduces successful fertility by 40%, and causes canceled cycle and implantation failure (Sharma et al., 2013). This narrative findings Advise to professional athletes, reduced intensity and duration of exercise to 3 times per week, for 45-60 minutes.

### 4.4 The Relationship of ART Results With Psychological Health

Infertility per se is stressful, and leads to social pressures induced by diagnosis, treatment, failure, unfulfilled wishes, and financial costs. Every stress appears to affect sperm concentration, content and morphology, and through reduction in testosterone and LH, gonad function is impaired and sperm parameters are changed. Increased adrenergic activity due to stress causes testicular vasoconstriction, resulting in lower testosterone and spermatogenesis (Anderson, Nisenblat, & Norman, 2010; Sharma et al., 2013). Through impairment of hypothalamic function, stress can cause both changes in neurotransmitters and catecholamine, and impair hypothalamic receptors because level of hormones such as progesterone, cortisol, norendocrine, and prolactin changes, and each has different negative impacts on fertility. This may also cause sexual dysfunction in men and women alike, which is conducive to infertility (Klonoff-Cohen, 2005). Stress, sexual dysfunction, and treatment failure can cause anxiety and ultimately lead to depression and increase likelihood of smoking and alcohol and substance use in these people (Anderson et al., 2010). Women working in excess of 32 hours per week have a long wait for pregnancy compared to those that work between 16 hours and 32 hours weekly. There are reasons for greater success rate of fertility by 54% in women that have received cognitive behavioral interventions compared to those that have not. Depression and anxiety decreased in women that received counseling, and chances of pregnancy increased in them. According to a report, amylase (not cortisol or adrenaline) is negatively correlated with fertility; although its mechanism is not clear, there is a theory that catecholamines can restrict blood flow into fallopian tube (Ando et al., 2013; Baldur-Felskov et al., 2013; Nakao et al., 2011). Various studies have concluded that depression and anxiety reduced in women that received counseling during infertility treatment, and their chances of pregnancy greatly increased, so that giving live birth is associated with having a positive attitude, conversely, anxiety increases chances of stillbirth (Sharma et al., 2013; Twigt et al., 2012). Problem-solving skills, coping with negative mood and anxiety, cognitive-behavioral interventions can be appropriate interventions.

### 4.5 The Relationship of ART Outcomes With Avoiding Medication, Drugs and Alcohol

Smoking increases amounts of ROS and semen leukocytes. Moreover, endocrine function may be affected by smoking, so that LH and FSH levels are reduced, resulting in reduced testosterone. Smoking may also affect ovarian reserves. Women that smoke 10 cigarettes per day experience an increase in urinary FSH, and with 20 cigarettes, they experience a reduction in progesterone in luteal phase. Furthermore, chemicals in cigarette smoke may slow down transfer of fertilized eggs from tube, and thus increase chances of ectopic pregnancy (Sharma et al., 2013).

Some lung diseases, such as asthma are already aggravated by pregnancy, and if these people also smoke, their asthma is further aggravated, resulting in hypoxia and lack of intrauterine growth (IUGR), and fetal and neonatal mortality is also increased in them (Newman, et al., 2010). People that were exposed to cigarette smoke in childhood and adolescence are twice more likely to develop lung cancer, compared to other people. If a pregnant woman is exposed to cigarette smoke, which affects germinal and embryonic cells, the fetus is exposed to future risk of cancer (Ortega-Garcia et al., 2010).

Toxic substances like cadmium and cotinine in cigarettes increase oxidative stress (OS), and consequently affect quality of oocytes, fertilization rate and quality of embryo. In fact, antioxidants protect oocytes against harm. Thus, oocytes will be more at risk if antioxidants are reduced (Donadini, Spalla, & Beone, 2008; Kazemi et al., 2013; Paszkowski, Clarke, & Hornstein, 2002; Vals et al., 2013). Having a healthy lifestyle leads to health in every dimension of life. To reduce health care costs, it is recommended that people be taught to change their behavior and lifestyles (Vals et al., 2013). Application of teaching healthy lifestyle has been found highly effective in changing people’s lifestyles (Greil, McQuillan, Lowry, & Shreffler, 2011; Lali et al., 2012). Furthermore, people exposed to cigarette smoke (smoker spouses) also develop many complications. Contact with cigarette smoke includes inhaling smoke from burning cigarette, and inhaling expiratory smoke from a smoker. Smoking at home comprise 71% of women’s contact with cigarette smoke. Women with smoker spouses are exposed to an average 4.2 hour of cigarette smoke daily (Ma, 2007).

In America, 50% and in Iran, 32.75% of women are exposed to cigarette smoke, and level of toxic substances in their body is 30%-40% of that of heavy smokers, which causes reduced ovarian reserves, ovarian corpus luteum and vascular changes, and directly affects steroidogenesis and gametogenesis, resulting in early menopause, delayed fertility by more than 6 months, reduced steroids and aromatization and increased chances of poor response to ovulation (Benedict et al., 2011; Chen et al., 2005; Kazemi et al., 2009; Soldin, Makambi, Soldin, & O’Mara, 2011). Other hormones like PRL and TSH also affect fertility, and in these people, level of serum PRL increases, which adversely affect fertility health (Benedict, 2011; Benedict et al., 2012; Benedict et al., 2011).

In women, alcohol use impairs estrogen and progesterone levels, resulting in impaired ovulation, implantation and fertilization and blastocyst development (Anderson et al., 2010). Alcohol can reduce chances of pregnancy and implantation, and increase risk of immediate or early miscarriage and fetal death. It can also cause LPD and blastocyst abnormalities. Researchers believe that alcohol affects hormonal fluctuations, which increase estrogen leading to reduced FSH and inhibition of follicle production and ovulation, but the main mechanism has not yet been identified (Anderson et al., 2010; Sharma et al., 2013; Vals et al., 2013).

Caffeine is a nervous system stimulant, and its daily consumption cannot be measured because it exists in various drinks such as cocoa, coffee, medicines, chocolate, and energizers. Therefore, women seeking pregnancy should reduce their daily intake of caffeine to 110-200 mg, or fewer than 2 cups per day. Excessive intake of caffeine, more than 5 cups per day, can increase paraxanthine and miscarriage. It has been reported that caffeine is associated with a long wait for pregnancy of 5-9 months, especially, with intakes more than 500 mg per day. Its negative impacts include: early miscarriage, fetal death, and stillbirth (Anderson et al., 2010; Cunningham et al., 2010; Klonoff-Cohen, 2005; Sharma et al., 2013).

Marijuana is the most common drug among teenagers. Through central and peripheral effects, this substance can cause harmful effects on pregnancy, which include production of cannabinoids that bind to uterine structure, cavities and tracts, and in men, reduced testosterone, sperm mobility and acrosome reaction capacity. In women, it can cause miscarriage, short-term reduction in LH, but long-term tolerance, which may have negative effects on tube movement, impair development of placenta and fetus leading to stillbirth (Sharma et al., 2013). Leflonomide that is used in treatment of rheumatoid arthritis, should not be used 2 years before and during pregnancy, nor should anti-cancer drugs, such as cyclophosphamide that kill cells or cause DNA deformation (Cunningham et al., 2010).

Cocaine has central and peripheral effects, and causes vasoconstriction and numbness, and changes mood and behavior through reuptake of neurotransmitter substances. Its long-term use can reduce sexual arousal, and in men, can cause delayed or failed erection and orgasm and reduced free testosterone level. Its destructive effects are associated with duration and dosage of use and concurrent use with other medications. In women, it can damage receptors, and cause placental abruption due to vasoconstriction. Opioids, like methadone and heroin, cause depression and reduce pain. In men, opioids cause sexual dysfunction and reduced semen parameters by affecting neurotransmitters, and in women, cause placental abruption and infertility (Cunningham et al., 2010; Sadock, 2007; Sharma et al., 2013).

### 4.6 The Relationship of ART Outcomes with Disease Prevention

Following tetanus, measles, and influenza vaccinations, presence of antibodies in mother, ensures maternal and fetal health. Maternal measles infection during pregnancy increases chances of miscarriage or fetal abnormalities (Anderson et al., 2010). To prevent non-communicable diseases in women with diabetes, or in those with a history of gestational diabetes in previous pregnancies, it is essential to control HbA1c before pregnancy and in the first trimester, as well as blood sugar level, for a healthy and low-risk pregnancy. Avoiding illicit relationships is advised to prevent sexually transmitted infections, which reduce PID incidence, and ultimately increase chances of pregnancy (Ashok Agarwal et al., 2012; Cunningham et al., 2010; Gallegos-Avila, Ancer-Rodríguez, Ortega-Martínez, & Jaramillo-Rangel, 2010; Hu & Willett, 2002). Timely vaccination and avoiding high-risk sexual behaviors are benefit for preventing disease.

### 4.7 The Relationship of ART Outcomes with Environmental Health

The effects of substances like methyl-mercury, pesticides, welding fumes, organic solvents, radioactive materials, and household adhesives have proved hazardous for pregnancy outcomes (Anderson, Nisenblat, et al., 2010). In men, contact with some substances may leave residue in semen for as long as 2 months, and cause abnormality, disorder, and chromosomal mutations in their children. These substances include ethanol, cyclophosphamide, lead, opioids, mercury, anesthetic gases, and hydrocarbons, which cause early miscarriage. Higher rates of pre-term labor, stillbirth, and restricted growth have been seen in men and women working in industrial and textile factories. Also, more abnormalities have been seen in children of those working in wood industry, fire departments, painters, and tea houses (Cunningham et al., 2010; Donadini et al., 2008; Kazemi et al., 2013). Avoiding exposure to chemicals, observing principles of safety, and wearing proper work uniform were recommended.

### 4.8 The Relationship of ART Outcomes with Spiritual Health

Spirituality is experiences that cannot be acquired through the five senses, but people discern it in their depth as their spiritual dimension. Human connects with spiritual dimension, with his God, and with people around. Thus, in this area, skills should be taught that reflect and strengthen spiritual aspect of promoting healthy life (Bolhari, 2012). Chronic diseases are the main cause of mortality. The long-term nature of chronic diseases changes treatment from elimination and eradication of disease to preserving function. Spiritual beliefs make it easier to accept negative psychological effects of chronic diseases, reduce depression, and increase satisfaction in life. According to research on AIDS patients, a positive and significant relationship was found between spirituality and quality of life, and a negative and significant relationship between spirituality and suffering. Strengthening spirituality increases psychosocial health, reduces suffering, and improves quality of patients’ lives. Moreover, since infertility treatment also follows a chronic and long process, and one of the reasons for treatment failure is discontinuation due to despair, people that enjoy higher spiritual health are expected to better withstand treatment (Litwinczuk & Groh, 2007; Versano, 2011).

There is a positive and significant relationship between mental health and resilience, and the same relationship exists between spiritual intelligence and resilience. Resilience helps people to endure hardship in life. Resilient people adapt well to environmental stressors. Positive and religious attitude support resilience (Meyer, Schwartz, & Frost, 2008).

Spirituality is largely related to low emotional distress and high quality of life, and poor religiosity is associated with distress, anxiety and lower quality of life. Thus, people with spiritual health and religious beliefs can better adapt to chronic diseases like cancer (Versano, 2011). Furthermore, spiritual development is recommended in textbook of spiritual intervention in counseling and psychotherapy, and ignoring religious beliefs has been seen to reduce the effect of counseling. It is argued that non-believers run greater risk of death (Miller, 2003; Post & Wade, 2009). Regard to review findings, researchers suggest considering spiritual care among infertile couple.

### 4.9 The Relationship of ART Outcomes With Social Health

Social skills are an important source of coping, which involve the ability to establish relationships with others through effective social connections. In relation to infertile couples, it has been observed that women are more inclined to talk about the issue than men, and men that do not disclose their infertility problem, enjoy lower well-being. Avoiding the truth about infertility is associated with poor coping (Schmidt, Holstein, Christensen, & Boivin, 2005).

Racial and sexual discriminations create lower social connections and greater tension. All perceived stresses in men and women are associated with their sociability level, and these people experience less stress if they are socially supported (Meyer et al., 2008).

Stress is disproportionately distributed among different social groups, including women, blacks, and economically deprived, and these people enjoy less stress support and compliance resources. Thus, they are exposed to greater psychological and social harms. It is argued that level of perceived stress in these people is similar to those in the process of divorce, losing job, or losing a dear one (Lee & Turney, 2012). Human fertility is affected by genetic and behavioral factors, and different levels of training can influence genetic factors, which include sociological intraction, demographic status, traditions, and other factors. As a result, social health has a special importance in fertility (Kohler, Rodgers, Miller, Skytthe, & Christensen, 2006).

Generally, there is a relationship between psychological health, social support and resilience. Social support from significant others such as family and friends can generate self-esteem, self confidence and ultimately, psychological health. More extensive social network helps people have higher mental health, cope with problems better, and find better solutions. Consequently, success rate of Assisted Reproductive Technique will also increase through reduction in level of stress (Sadock, 2007). This issue is highly important to infertility clinics that need to have comprehensive counseling programs for their patients, to properly teach them stress coping techniques, so that it is internalized like other behaviors because infertility is a chronically stressful factor that can affect every aspect of people’s lives, disrupt their relationship with others, and make them avoid socialization, leading to lower self-belief (Gillian Homan, Litt, & Norman, 2012). Teaching problem-solving skills, at high level, can be helpful, so that people do not just ignore the problem, but look for its causes, and find appropriate solutions through proper planning and correct techniques. It has been observed that people that had been trained consulted others more about their problems (Schmidt et al., 2005). Relatively brief interventions have led to dramatic improvements in infertile people. It has been recommended that infertility clinics prepare counseling interventions from various aspects for infertile couples (Schmidt, Tjørnhøj-Thomsen, Boivin, & Andersen, 2005). In addition, the effect of teaching healthy lifestyle on girls at entry to school, and its impact on their future infants’ health be emphasized. Trained women deliver healthier children (McCrary & Royer, 2006). Teaching communication skills and life skills, and problem-solving skills were benefit for social health in infertile couple that affects to infertility treatment outcomes.

## 5. Conclusion

In this article, different aspects of lifestyle and their relationship with infertility have been addressed. Review of literature showed that lifestyle includes physical, psychological, social factors, spiritual and is associated with people’s general and reproductive health. It is also clear that lifestyle is an important factor that can be altered. Thus, through assessment of lifestyle pattern of infertile couples, and healthy lifestyle counseling programs before and during ART, success rate of this technique can be significantly improved.

**Recommendations**

Infertility dramatically affects the family and society, treatment process can be long and stressful, and impose heavy financial burden, and hormonal drugs can be stressful for the patient due to risk of cancer, it is therefore recommended that there be a comprehensive healthy lifestyle counseling program in infertility treatment centers and healthy lifestyle training for various academic years in community health programs.
